# Comparison of selected exercise training modalities in the management of PCOS: A systematic review and meta-analysis to inform evidence-based guidelines

**DOI:** 10.1016/j.jsampl.2023.100024

**Published:** 2023-04-28

**Authors:** Giorgia E. Colombo, Xela Dafauce Bouzo, Rhiannon K. Patten, Aya Mousa, Chau Thien Tay, Loyal Pattuwage, Helena J. Teede, Leanne M. Redman, Angelica Lindén Hirschberg, Angelo Sabag

**Affiliations:** a Royal Infirmary of Edinburgh, Edinburgh, UK; b Department of Healthcare, Cardiff Metropolitan University, Cardiff, UK; c Institute for Health and Sport (iHeS), Victoria University, Melbourne, VIC, Australia; d Monash Centre for Health Research and Implementation (MCHRI), Monash University, Melbourne, VIC, Australia; e Pennington Biomedical Research Center, Louisiana State University, Baton Rouge, LA, USA; f Department of Women's and Children's Health, Karolinska Institutet and Department of Gynecology and Reproductive Medicine, Karolinska University Hospital, Stockholm, Sweden; g NICM Health Research Institute, Western Sydney University, Penrith, NSW, Australia; h Discipline of Exercise and Sport Science, Faculty of Medicine and Health, The University of Sydney, Sydney, NSW, Australia

**Keywords:** Polycystic ovary syndrome, Exercise, High intensity interval training, HIIT, Moderate intensity continuous training, MICT, Resistance training, RT, Cardiometabolic health

## Abstract

**Background:**

Polycystic ovary syndrome (PCOS) is a common endocrine condition in women of reproductive age that often presents with reproductive, metabolic, and psychological symptoms. While exercise is part of the management of PCOS, it is unclear which form of exercise may be most effective and for which outcomes.

**Aim:**

In order to inform the updated 2023 International evidence-based guideline for the assessment and management of polycystic ovary syndrome, this systematic review aimed to determine the exercise modality that provides the greatest improvement in anthropometric, metabolic, hormonal/reproductive, and psychological outcomes in adult women with PCOS.

**Methods:**

Five databases were searched from inception to July 2022. Studies eligible for inclusion consisted of those in a PCOS population, that compared two exercise modalities, and reported at least one anthropometric, metabolic, hormonal/reproductive, and/or psychological outcome. Screening, data extraction, and methodological quality assessments were conducted by two independent reviewers. Methodological quality assessment was performed using the Cochrane Risk of Bias tool and the Grading of Recommendations Assessment, Development and Evaluation (GRADE) guidelines were used to determine the certainty of evidence. Meta-analysis was performed utilising Comprehensive Meta-Analysis software, Version 3.

**Results:**

Of the 4739 records identified, five unique studies were eligible for inclusion in the systematic review and meta-analysis, comprising a total of 216 individuals. Meta-analyses comparing high-intensity interval training (HIIT) to moderate-intensity continuous training (MICT) on anthropometric, metabolic, and hormonal/reproductive parameters found no statistically significant differences in outcomes between groups, and the certainty of evidence was graded as low or very low. Results from single studies showed that HIIT was more effective than MICT for menstrual regularity (odds ratio [95% confidence interval] ​= ​7.875 [1.105, 56.125], p ​= ​0.039, very low certainty). HIIT vs resistance training, and diet ​+ ​MICT vs diet ​+ ​MICT ​+ ​resistance training were examined by a single study each, and no statistically significant differences were found for any outcome, with the certainty of evidence ranked as very low.

**Conclusion:**

To date, there are insufficient RCTs comparing exercise modalities in individuals with PCOS to establish with certainty whether one form of exercise is superior to another for the management of PCOS.

## 1 Introduction

Polycystic ovary syndrome (PCOS) is considered the most common endocrine condition affecting 2%–20% of women of reproductive age [1,2]. The disease encompasses reproductive, metabolic, and psychological symptoms, leading to reduced quality of life [3]. Diagnosis is typically based on the Rotterdam criteria, which requires two of the following to be present: oligo- or anovulation, hyperandrogenism, and polycystic ovaries [4]. Insulin resistance, hyperinsulinaemia, and obesity have also been shown to exacerbate the clinical manifestations of PCOS [3,5].

Lifestyle interventions, involving diet and exercise, are first-line therapies for the management of PCOS [6,7]. Among the general population, exercise is critical for prevention and treatment of chronic disease [8]. In individuals with PCOS, exercise improves cardiorespiratory fitness and lowers waist circumference [9], as well as increasing insulin sensitivity [7,10,11]. Current guidelines for the management of PCOS recommend a minimum of 150 ​min/week of moderate intensity exercise or 75 ​min/week of vigorous exercise, aiming for 30 active minutes daily [12]. Muscle strengthening activity is also recommended but no further prescription details are described [12]. While exercise is a broad all-encompassing word which includes any pre-planned and structured physical activity, common exercise modalities include resistance training (RT) and aerobic exercise, which can involve high-intensity interval training (HIIT) and/or moderate-intensity continuous training (MICT).

HIIT has been demonstrated to improve cardiometabolic health in populations at increased risk of cardiovascular disease, such as individuals with metabolic syndrome or type 2 diabetes mellitus [[13], [14], [15]], while requiring a lower energy expenditure and less time commitment [16]. In particular, improvements in insulin resistance and cardiorespiratory fitness (VO_2_max) may be superior with HIIT than traditional continuous training [13]. Meta-analyses have demonstrated greater improvements in cardiorespiratory fitness, an important indicator of cardiometabolic health [17,18], with HIIT than MICT in both a healthy population [19] and in patients with lifestyle-induced cardiometabolic disease [20]. A clinical study showed that HIIT and MICT, as well as RT, improved sexual function and psychological symptoms in individuals with PCOS [21].

Although the benefits of exercise per se in comparison to no exercise have been examined in the PCOS population in multiple systematic reviews and meta-analyses [5,8,9,22], there is a paucity of research comparing specific exercise modalities in the management of this condition. This review aimed to determine the exercise modality that provided the greatest improvement in anthropometric, metabolic, hormonal/reproductive, and psychological outcomes in a population with PCOS. This review directly informed the updated 2023 international evidence-based guidelines for the assessment and management of PCOS [23].

## 2 Materials and methods

This systematic review is an update of a review prepared to inform clinical practice recommendations in the National Health and Medical Research Council approved International evidence-based guideline for the assessment and management of polycystic ovary syndrome [23]. The clinical question posed in this systematic review is: in women with PCOS, are exercise interventions (compared to different exercises) effective for improving anthropometric, metabolic, reproductive, fertility, quality of life and emotional wellbeing outcomes?

### 2.1 Literature search strategy

A comprehensive search of online databases for articles relevant to the review was conducted. The following databases were searched from inception to July 2022: Medline (Ovid), PsycInfo, EMBASE, All EBM, and CINAHL. The search algorithm consisted of terms related to polycystic ovary syndrome, anovulation, oligo-ovulation, hyperandrogenism, exercise, resistance training, aerobic exercise, and endurance training; truncations were utilised, and search terms were adapted to various databases as appropriate. The full search strategy is reported in Appendix 1.

Only randomised trials, published in English, were eligible for inclusion.

### 2.2 Inclusion and exclusion criteria

#### 2.2.1 Population

Eligible studies considered a population of individuals of the female sex diagnosed with PCOS. No limitations were placed on age, ethnicity, weight, or other co-morbidities. The Rotterdam 2003 diagnostic criteria [4], National Institute of Health 1990 diagnostic criteria [24], and Androgen Excess and PCOS Society 2006 criteria [25] were all accepted as diagnostic methods. Exclusion criteria included individuals without PCOS, those taking anti-obesity medications, and patients that had undergone bariatric surgery.

#### 2.2.2 Intervention

Eligible studies employed an intervention consisting of any type of quantifiable exercise regime that documented the type, intensity, frequency, and duration of exercise. Exclusion criteria included studies in which the primary intervention component or control arm was a medication to manage clinical or metabolic features of PCOS, unquantifiable exercise interventions, and exercise interventions used in conjunction with anti-obesity medications.

#### 2.2.3 Comparator

Eligible studies compared an exercise intervention to another exercise intervention (see 2.2.2). Studies comparing an exercise intervention to a control group not following an exercise regime were excluded (e.g. diet and exercise vs diet only).

#### 2.2.4 Outcome

Eligible studies reported changes in any of the following sets of outcomes: (a) anthropometric measures including weight, body mass index (BMI), and waist circumference (WC); (b) metabolic factors including HbA1c, fasting insulin, fasting glucose, homeostatic model assessment of insulin resistance (HOMA-IR), oral glucose tolerance test (OGTT) insulin, OGTT glucose, lipids (triglycerides, low-density lipoprotein (LDL), high-density lipoprotein (HDL), and/or total cholesterol), and systolic blood pressure (SBP); (c) hormonal or reproductive parameters including clinical hirsutism, biochemical hyperandrogenism (HA), total testosterone, free testosterone, sex hormone binding globulin (SHBG), free androgen index (FAI), ovulation, and menstrual regularity; and (d) psychological outcomes including anxiety, depression, and quality of life. Units of measurement were standardised for meta-analysis.

### 2.3 Study selection

The results of the database searches were collated, duplicates were removed, and studies were screened by two independent reviewers (AS, RKP, GEC, or XDB). Screening was performed based on eligibility criteria, initially by title and abstract, and then by full-text. Where disagreement occurred, consensus was achieved via discussion and input from a third reviewer (RKP, GEC, or XDB).

### 2.4 Data synthesis

The data extracted reflected outcomes outlined in the PICO framework. Data regarding participant characteristics (age, BMI, and PCOS diagnostic criteria), exercise interventions (mode, frequency, intensity, session duration, intervention duration), additional interventions (dietary and/or pharmacological prescriptions), and pre- and post-intervention measures were extracted. The data extraction was performed by two individual reviewers (AS and XDB) and conflicts were resolved via consensus. Attempts were made to contact authors when study information was missing. After two attempts to contact an author with no response, the respective study was not pooled in meta-analysis.

### 2.5 Data analysis

Where appropriate (>1 study available), data were pooled, and meta-analysis was performed utilising Comprehensive Meta-Analysis, Version 3 (Biostat Inc., Englewood, NJ, USA) [26]. Random effects models were used, which assume a degree of clinical heterogeneity between studies, and the weighted mean differences with 95% confidence intervals (CIs) were assessed. Cochrane's Q and I^2^ statistics were used to quantify statistical heterogeneity between studies. Subgroup analysis was based on exercise type (HIIT, MICT, or RT), comparing the effects of each modality on anthropometric, metabolic, hormonal/reproductive, and psychological outcomes.

### 2.6 Methodological quality assessment

Risk of bias was assessed by two authors (AS and GEC) using the Cochrane Collaboration's Risk of Bias tool [27], which comprises six categories of bias: selection, performance, detection, attrition, reporting, and other bias. Each category was characterised as either: low risk of bias, high risk of bias, or unclear risk of bias. The certainty of evidence for each outcome was summarised and scored according to the Grading of Recommendations Assessment, Development and Evaluation (GRADE) guidelines [28].

## 3 Results

**Fig. 1:**
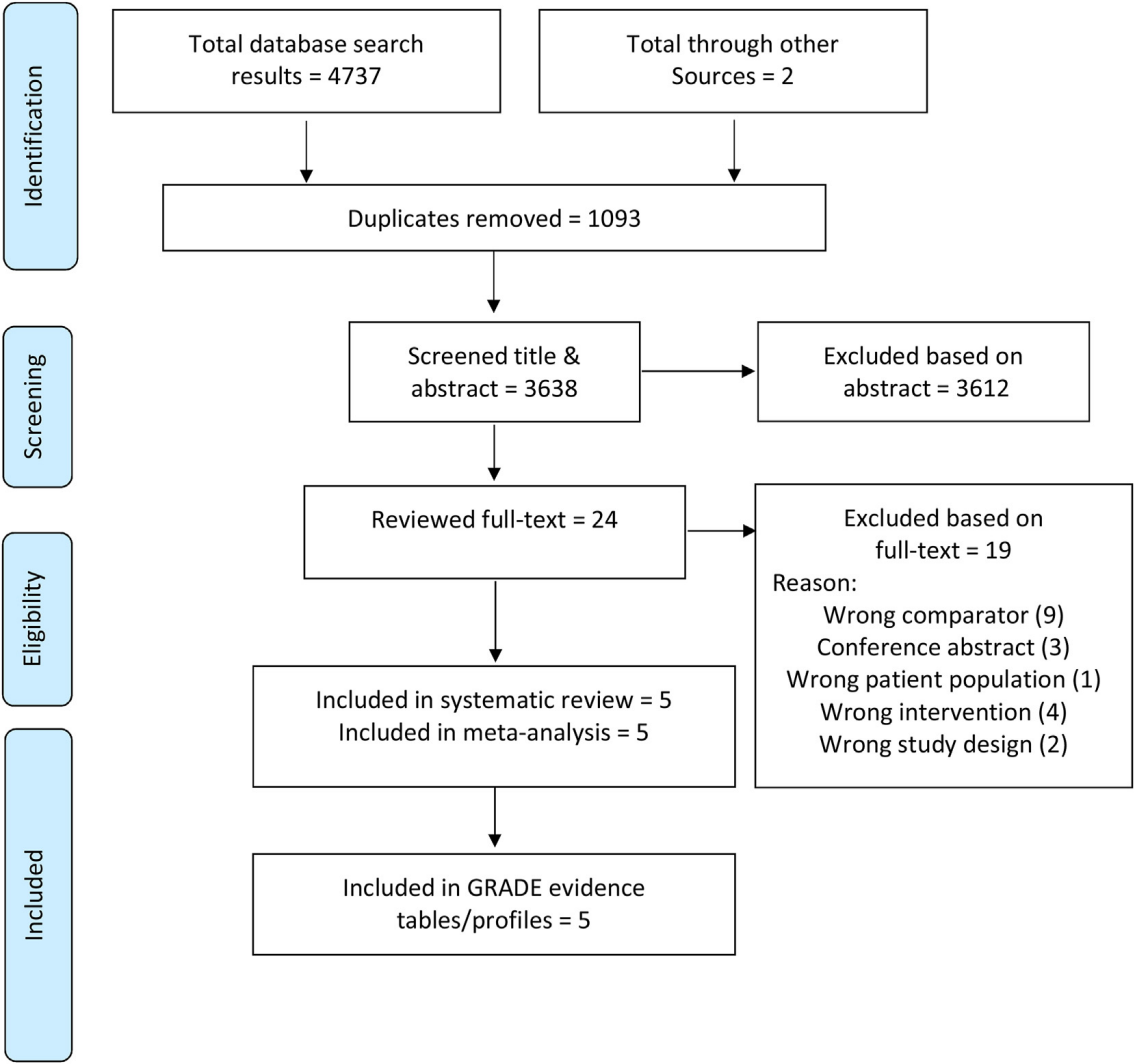
Preferred Reporting Items for Systematic Reviews and Meta-analyses (PRISMA) flow diagram.

The screening strategy identified 4739 records, of which five unique were included in this systematic review (Fig. 1). A table of the studies excluded at the full-text review stage with reasons for exclusion is provided in Appendix 1.

### 3.1 Participant characteristics

The five unique studies included in this systematic review comprised a total of 216 individuals, 76 in HIIT groups, 96 in MICT groups, 11 in an RT group, and 33 in a MICT ​+ ​RT group. The mean age ranged from 29.0 to 32.5 years, and the mean BMI ranged from 26.1 to 38.4 ​kg/m^2^. All studies diagnosed PCOS according to the Rotterdam consensus criteria [4].

### 3.2 Intervention characteristics

Three studies compared HIIT to MICT, one study compared HIIT to RT, and one study compared diet plus MICT and RT versus diet plus MICT only. Intervention duration varied from ten weeks to six months. Most exercise interventions complied with, at least, part of the current guideline on physical activity for the management of PCOS [12]. Patten et al., 2022 [29] and Ribeiro et al., 2020 [30] calculated training volume and matched this across intervention groups. Study characteristics are summarised in Table 1.

**Table 1:** Study characteristics.

Author, year, country	Population/Setting	Study Design	Sample Size	Intervention /exposure details	Comparison/control details	Follow up Duration	Mean Age (±SD)	Mean BMI (±SD)	Outcomes	Summary of findings
Almenning 2015 Norway	Inactive^a^ women with PCOS	Parallel RCT	HIIT: 10 RT: 11	HIIT 3 days per week	RT 3 days per week	10 weeks	27.2 ​± ​5.5	HIIT: 26.1 ​± ​6.5 RT: 27.4 ​± ​6.9	Metabolic, cardiovascular, and hormonal outcomes	HIIT improved insulin resistance, body composition. RT improved body composition
Benham 2021 Canada	Inactive^a^ women with PCOS	Parallel RCT	HIIT: 16 MICT: 14	HIIT 3 days per week	MICT 3 days per week	6 months	HIIT: 29.1 ​± ​4.1 MICT: 29.5 ​± ​4.6	HIIT: 31.4 ​± ​8.6 MICT: 31.3 ​± ​9.0	Reproductive, anthropometric and cardiometabolic outcomes	MICT and HIIT were both effective at improving anthropometrics and some cardiometabolic health markers.
Patten 2022 Australia	Inactive^a^ women with PCOS	Parallel RCT	HIIT: 15 MICT: 14	HIIT 3 days per week	MICT 3 days per week	12 weeks	HIIT: 29.7 ​± ​4.8 MICT: 32.5 ​± ​6.2	HIIT: 35.5 ​± ​6.8 MICT: 38.4 ​± ​9.3	Insulin sensitivity, hormonal profiles, menstrual cyclicity and body composition.	HIIT offers greater improvements in aerobic capacity, insulin sensitivity and menstrual cyclicity, and larger reductions in hyperandrogenism compared to MICT
Ribeiro 2020 Brazil	Sedentary women with PCOS	Parallel RCT	HIIT: 35 MICT: 37	HIIT 3 days per week	MICT 3 days per week	16 weeks	HIIT: 29.0 ​± ​4.3 MICT: 29.1 ​± ​5.3	HIIT: 28.7 ​± ​4.8 MICT: 28.4 ​± ​5.6	Hormonal, metabolic, anthropometric, quality of life, depression and anxiety.	MICT and HIIT training improved hormonal, anthropometric, anxiety and depression, and quality of life. Only HIIT training reduced the FAI. Only MICT training improved lipid profile.
Thomson 2008 Australia	Inactive^a^ women with PCOS	Parallel RCT	MICT: 31 MICT ​+ ​RT: 33	MICT 5 days per week	MICT 3 days per week and RT 2 days per week	20 weeks	29.3 ​± ​6.8	36.1 ​± ​4.8	Weight, body composition, cardiometabolic risk factors, hormonal status, menstrual cyclicity, and ovulatory function.	The addition of aerobic or combined aerobic resistance exercise to an energy-restricted diet improved body composition but had no additional effect on improvements in cardiometabolic, hormonal, and reproductive outcomes relative to diet alone.

SD: standard deviation; RCT: randomised controlled trial; HIIT: high-intensity interval training; MICT: moderate-intensity continuous training; RT: resistance training.

a Participants were excluded if they were performing regular physical exercise.

Studies involving HIIT interventions included varied HIIT approaches. Almenning et al., 2015 [31] prescribed twice weekly sessions of four 4-min intervals at 90–95% HRmax, separated by 3 ​min of moderate-intensity exercise at 70% of HRmax; and one weekly session of ten 1-min intervals at maximal intensity (‘all out’), separated by 1-min rest/very low activity. Benham et al., 2021 [32] implemented ten cycles of 30 ​s at high intensity (90% of heart rate reserve, or 9/10 on a modified Borg scale), alternating with 90 ​s of low-intensity aerobic exercise. Patten et al., 2022 prescribed twice-weekly sessions of twelve 1-min intervals at 90–100% peak heart rate (HRpeak), separated by 1 ​min of active recovery and one weekly session of eight 4-min intervals at 90–95% HRpeak, interspersed with a 2-min active recovery. Finally, Ribeiro et al., 2020 [30] prescribed twice-weekly sessions of six to ten 2-min intervals at 70–90% of the HRmax interspersed with 3-min recovery periods.

MICT interventions were more consistent. Participants in Benham et al., 2021 [32] completed 40 ​min of moderate-intensity aerobic exercise (50%–60% HRmax, or 4–6/10 on a modified Borg scale), whereas MICT in Thomson et al., 2008 [7] consisted of walking or jogging five times per week for 25–45 ​min at 60–80% HRmax. In Patten et al., 2022 [29], participants completed three sessions per week of 45 ​min of continuous cycling at 60–75% HRpeak, and comparably MICT in Ribeiro et al., 2020 [30] involved thrice-weekly sessions of 30–45 ​min of continuous cycling at 65–80% HRmax.

For RT, Almenning et al., 2015 [31] prescribed eight dynamic exercises at 75% of their one repetition maximum, with three sets of ten repetitions separated by 1 ​min of rest between the sets. Thomson et al., 2008 [7] had a group completing MICT three days per week combined with two days of RT involving five exercises for three sets of 12 repetitions at 50–75% one-repetition maximum. The latter also included a concurrent treatment: an energy-restricted, high-protein diet (5000–6000 ​kJ/d) for a planned weight loss of 8–12 ​kg over the study period. This regimen was prescribed to all study participants in both the MICT and MICT plus RT groups [7].

Adherence rates were reported in all but one study [7] and were calculated as the number of sessions attended divided by the total number of scheduled sessions, reported as a percentage. Participants in Almenning et al., 2015 [31] had supervised exercise sessions once weekly, participants in Benham et al., 2021 [32] were supervised twice weekly, and in Patten et al., 2022 [29] and Ribeiro et al., 2020 [30], all exercise sessions were supervised by an exercise professional. The latter [30] reported 97.6% adherence in the HIIT group, compared to 85% in the MICT group. In Benham et al., 2021 [32], adherence was 81% (interquartile range (IQR) 56%, 85%) in the MICT group and 65% (IQR 51%, 85%; p ​= ​0.91) in the HIIT group. Patten et al., 2022 [29] found an adherence of 94% (±3.0%) in the HIIT group and 92% (±4.8%) in the MICT group. Almenning et al., 2015 [31] reported 90% adherence in the HIIT group and 87% adherence in the RT group. Thomson et al., 2008 [7] did not report adherence rates.

### 3.3 Meta-analysis

**Fig. 2:**
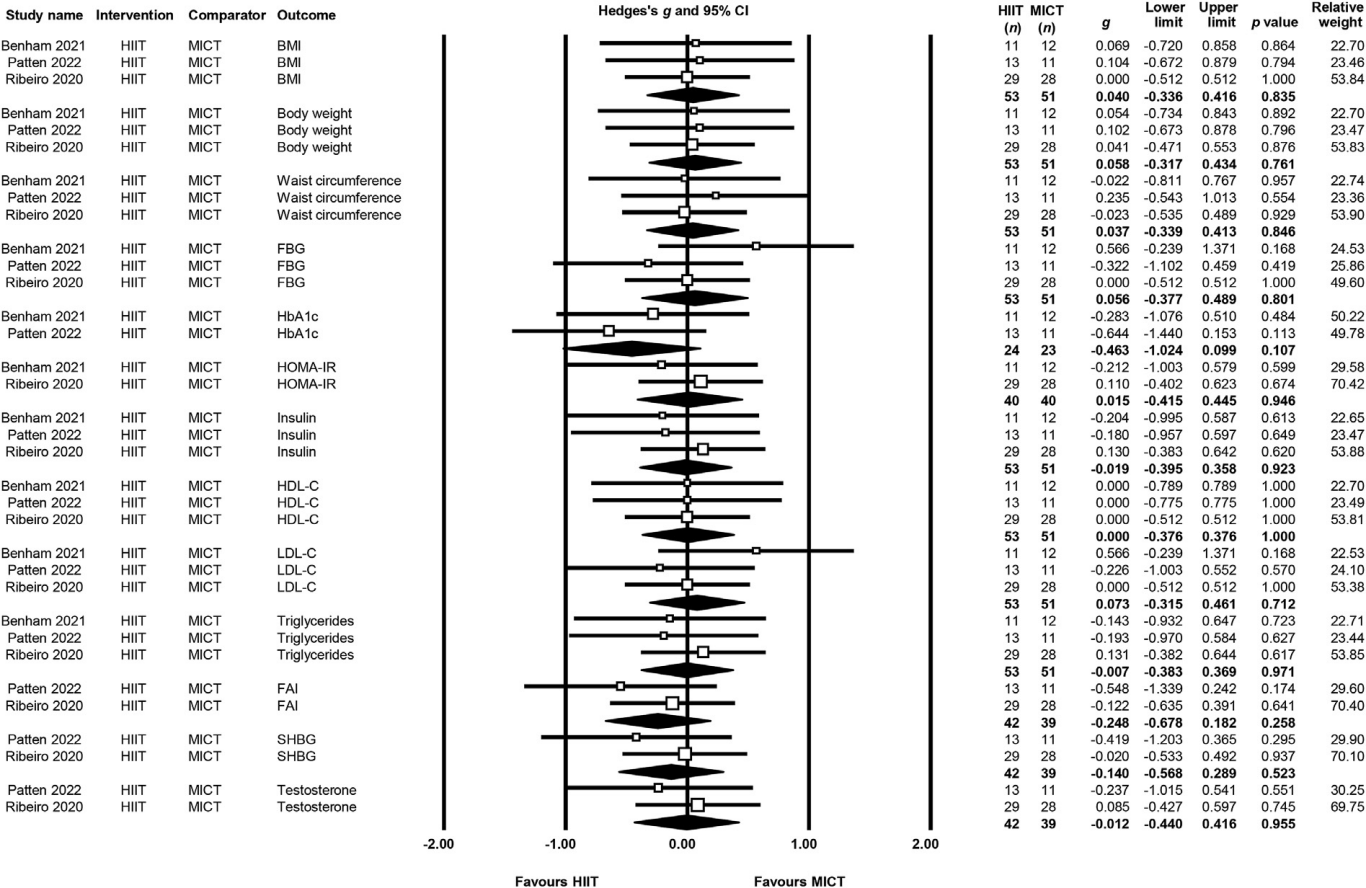
Forest plot for high-intensity interval training versus moderate-intensity continuous training.

Meta-analysis was performed on a total of three studies [29,30,32] comparing HIIT versus MICT for a range of anthropometric, metabolic, and hormonal/reproductive outcomes. There were no statistically significant differences in any of the outcomes assessed, with most of the evidence being of low to very low certainty due primarily to imprecision (small sample sizes), in addition to unclear risk of bias and inconsistency of effect estimates and/or confidence intervals. The outcomes analysed are presented in Table 2, and a forest plot for these outcomes is presented in Fig. 2. The comparisons of HIIT vs RT and diet ​+ ​MICT vs diet ​+ ​MICT ​+ ​RT were reported by a single study each and were therefore not amenable to meta-analysis, but are described narratively.

**Table 2:** Analysis of high-intensity interval training versus moderate-intensity continuous training [29,30,32].

Outcome	MD	95% confidence interval		p value	Favours	*I*^2^	***τ***	No. studies	HIIT (n)	MICT (n)	GRADE certainty
BMI (kg/m^2^)	0.186	−2.173	2.546	0.877	MICT	0	0	3	53	51	⊕⊕◯◯ Low
Body weight (kg)	1.058	−6.279	8.396	0.777	MICT	0	0	3	53	51	⊕⊕◯◯ Low
WC (cm)	0.378	−5.194	5.950	0.894	MICT	0	0	3	53	51	⊕⊕◯◯ Low
HbA1c (%)	−0.160	−0.336	0.017	0.076	HIIT	0	0	2	24	23	⊕⊕◯◯ Low
FBG (mmol/L)	0.053	−0.132	0.238	0.574	MICT	0	0	3	53	51	⊕◯◯◯ Very Low
Fasting Insulin (μIU/L)	0.352	−3.201	3.904	0.846	MICT	0	0	3	53	51	⊕◯◯◯ Very Low
HOMA-IR	−0.033	−0.742	0.675	0.926	HIIT	0	0	2	40	40	⊕◯◯◯ Very Low
HDL-C (mmol/L)	0.000	−0.111	0.111	1.000	No difference	0	0	3	53	51	⊕⊕◯◯ Low
LDL-C (mmol/L)	0.081	−0.125	0.288	0.441	MICT	0	0	3	53	51	⊕◯◯◯ Very Low
Triglycerides (mmol/L)	−0.049	−0.356	0.259	0.756	HIIT	0	0	3	53	51	⊕⊕◯◯ Low
SBP (mmHg)	−2.900	−6.042	0.242	0.070	MICT	0	0	1	11	12	⊕◯◯◯ Very Low
FAI	−1.677	−4.059	0.704	0.167	HIIT	0	0	2	42	39	⊕⊕◯◯ Low
SHBG (mmol/L)	6.324	−5.966	18.614	0.313	HIIT	0	0	2	42	39	⊕⊕◯◯ Low
Testosterone (nmol/L)	−0.086	−0.601	0.430	0.745	HIIT	0	0	2	42	39	⊕◯◯◯ Very Low
Menstrual regularity	OR 7.875	1.105	56.125	**0.039**	HIIT	0	0	1	13	11	⊕◯◯◯ Very Low
Anxiety	−0.300	−2.38	1.780	0.777	HIIT	0	0	1	22	23	⊕◯◯◯ Very Low
Depression	−0.700	−2.857	1.457	0.504	MICT	0	0	1	22	23	⊕◯◯◯ Very Low
Quality of life	SMD −0.278	−0.792	0.237	0.291	HIIT	0	0	1	29	28	⊕◯◯◯ Very Low

MD, mean difference; OR, odds ratio; SMD, standardised mean difference; HIIT, high-intensity interval training; MICT, moderate-intensity training; BMI, body mass index; WC, waist circumference; FBG, fasting blood glucose; HOMA-IR, homeostatic model assessment of insulin resistance; HDL, high-density lipoprotein cholesterol; LDL, low-density lipoprotein cholesterol; SBP, systolic blood pressure; FAI, free androgen index; SHBG, sex hormone-binding globulin.

### 3.4 Descriptive analysis

#### 3.4.1 HIIT vs MICT

In the single-study analysis (studies/outcomes not pooled in meta-analysis) of HIIT vs MICT, the outcomes examined included SBP, menstrual regularity, anxiety, depression, and quality of life. HIIT was more effective than MICT for menstrual regularity (odds ratio (OR) [95%CI] ​= ​7.875 [1.105, 56.125], p ​= ​0.039) with very low certainty of evidence due to imprecision (being derived from a single small study – Patten et al., 2022 [29]) as well as inconsistency and risk of bias. Menstrual cycle regularity was also reported by Benham et al., 2021 [32] as the percentage of participants with regular cycles. However, as no sample size was provided, these data could not be pooled in a meta-analysis, nor could the OR be determined. In this study, HIIT improved menstrual regularity from 50 to 53% of participants (p ​= ​0.85), and MICT improved menstrual regularity from 29 to 42% of participants (p ​= ​0.48). There were no statistically significant differences in the other outcomes assessed.

#### 3.4.2 HIIT vs RT

One study [31] compared HIIT with RT on the following anthropometric, metabolic and hormonal/reproductive outcomes: BMI, body weight, WC, FBG, fasting insulin, HOMA-IR, HDL-C, LDL-C, triglycerides, FAI, SHBG, and testosterone. There were no statistically significant differences between HIIT and RT for any outcomes. Certainty in these results is very low due to being derived from a single, relatively small study with an unclear risk of bias. These results are presented in Table 3.

**Table 3:** Analysis for high-intensity interval training versus resistance training [31].

Outcome	WMD	95% confidence interval		p value	Favours	*I*^2^	***τ***	No. studies	HIIT (n)	RT (n)	GRADE certainty
BMI (kg/m^2^)	−0.300	−5.824	5.224	0.915	HIIT	0	0	1	8	8	⊕◯◯◯ Very Low
Body weight (kg)	−0.900	−18.141	16.341	0.919	HIIT	0	0	1	8	8	⊕◯◯◯ Very Low
WC (cm)	−1.800	−16.569	12.969	0.811	RT	0	0	1	8	8	⊕◯◯◯ Very Low
FBG (mmol/L)	−0.100	−0.402	0.202	0.516	HIIT	0	0	1	8	8	⊕◯◯◯ Very Low
Fasting Insulin (μIU/L)	−1.700	−8.156	4.756	0.606	HIIT	0	0	1	8	8	⊕◯◯◯ Very Low
HOMA-IR	−0.600	−2.063	0.863	0.422	HIIT	0	0	1	8	8	⊕◯◯◯ Very Low
HDL-C (mmol/L)	−0.300	−0.749	0.149	0.190	HIIT	0	0	1	8	8	⊕◯◯◯ Very Low
LDL-C (mmol/L)	−0.400	−1.165	−0.365	0.306	RT	0	0	1	8	8	⊕◯◯◯ Very Low
TG (mmol/L)	−1.000	−0.619	0.419	0.705	HIIT	0	0	1	8	8	⊕◯◯◯ Very Low
FAI	−1.100	−2.610	0.410	0.153	RT	0	0	1	8	8	⊕◯◯◯ Very Low
SHBG (mmol/L)	31.600	−46.981	110.181	0.431	RT	0	0	1	8	8	⊕◯◯◯ Very Low
Testosterone (nmol/L)	−0.200	−1.002	0.602	0.625	RT	0	0	1	8	8	⊕◯◯◯ Very Low

MD, mean difference; HIIT, high-intensity interval training; RT, resistance training; WC, waist circumference; FBG, fasting blood glucose; HOMA-IR, homeostatic model assessment of insulin resistance; HDL, high-density lipoprotein cholesterol; LDL, low-density lipoprotein cholesterol; FAI, free androgen index; SHBG, sex hormone-binding globulin.

#### 3.4.3 Diet ​+ ​MICT vs diet ​+ ​MICT ​+ ​RT

One study [7] compared diet plus combined MICT and RT with diet and MICT on the following anthropometric, metabolic and hormonal/reproductive outcomes: body weight, WC, FBG, fasting insulin, HOMA-IR, HDL-C, LDL-C, triglycerides, SBP, FAI, SHBG, and testosterone. There were no statistically significant differences between diet plus combined MICT and RT and diet plus MICT for any of the outcomes. Certainty in these results is very low because they are derived from a single, relatively small study with a high risk of bias due to lack of blinding of outcome assessors, concealment of allocation (opaque envelopes), high dropout rate, and lack of clarity regarding whether analyses were undertaken as per-protocol or as intention-to-treat. These results are presented in Table 4.

**Table 4:** Analysis for diet plus combined aerobic and resistance training versus diet plus aerobic exercise [7].

Outcome	MD	95% confidence interval		p value	Favours	*I*^2^	***τ***	No. studies	HIIT (n)	RT (n)	GRADE certainty
Body weight (kg)	−1.500	−13.217	10.217	0.802	D ​+ ​AEx	0	0	1	20	18	⊕◯◯◯ Very Low
WC (cm)	−0.700	−8.901	7.501	0.867	D ​+ ​CT	0	0	1	20	18	⊕◯◯◯ Very Low
FBG (mmol/L)	−0.100	−0.453	0.253	0.579	D ​+ ​AEx	0	0	1	20	18	⊕◯◯◯ Very Low
Fasting Insulin (μIU/L)	−1.300	−6.919	4.319	0.650	D ​+ ​CT	0	0	1	20	18	⊕◯◯◯ Very Low
HOMA-IR	−0.160	−0.846	0.526	0.648	D ​+ ​CT	0	0	1	20	18	⊕◯◯◯ Very Low
HDL-C (mmol/L)	0.000	−0.166	0.166	1.000	No difference	0	0	1	20	18	⊕◯◯◯ Very Low
LDL-C (mmol/L)	−0.010	−0.580	0.560	0.973	D ​+ ​AEx	0	0	1	20	18	⊕◯◯◯ Very Low
Triglycerides (mmol/L)	−0.180	−0.771	0.411	0.550	D ​+ ​CT	0	0	1	20	18	⊕◯◯◯ Very Low
SBP (mmHg)	−3.100	−11.233	5.033	0.455	D ​+ ​CT	0	0	1	20	18	⊕◯◯◯ Very Low
FAI	−0.300	−4.196	3.596	0.880	D ​+ ​CT	0	0	1	20	18	⊕◯◯◯ Very Low
SHBG (mmol/L)	3.100	−7.471	13.671	0.565	D ​+ ​CT	0	0	1	20	18	⊕◯◯◯ Very Low
Testosterone (nmol/L)	−0.250	−0.755	0.255	0.332	D ​+ ​AEx	0	0	1	20	18	⊕◯◯◯ Very Low

MD, mean difference; D ​+ ​AEx, diet plus aerobic exercise; D ​+ ​CT, diet plus aerobic and resistance training; H HOMA-IR, homeostatic model assessment of insulin resistance; HDL, high-density lipoprotein cholesterol; LDL, low-density lipoprotein cholesterol; SBP, systolic blood pressure; FAI, free androgen index; SHBG, sex hormone-binding globulin.

### 3.5 Methodological quality and certainty of evidence

**Fig. 3:**
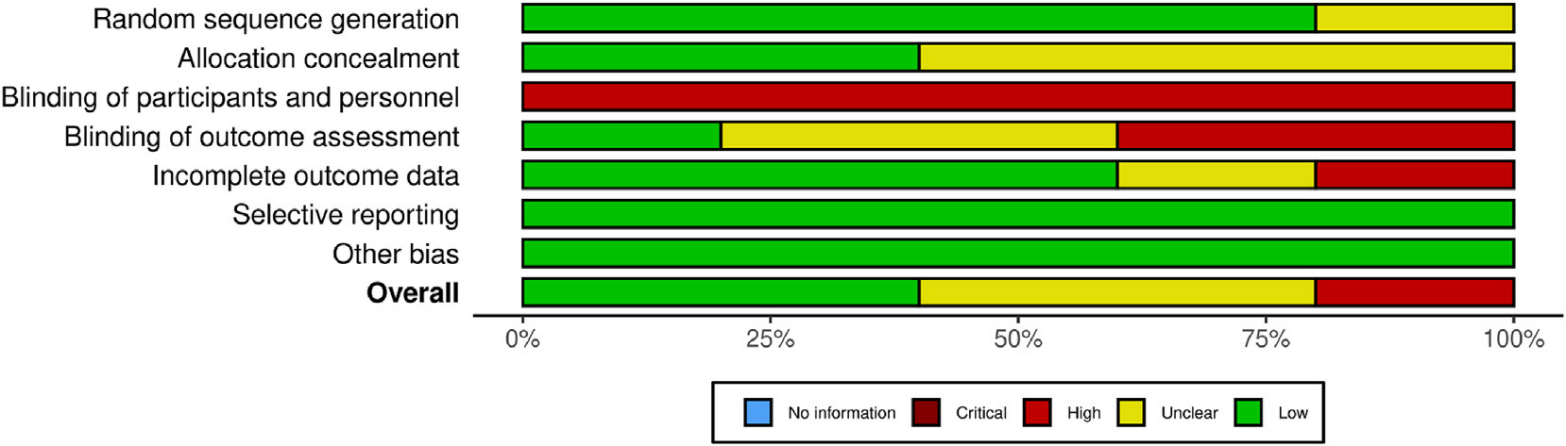
Risk of bias summary.

The risk of bias assessment found two studies at a low risk of bias [29,32], two studies with an unclear risk of bias [30,31], and one study at a high risk of bias [7]. All studies were ranked as high risk of bias for the category “Blinding of participants and personnel” due to a lack of blinding to participant group allocation by outcome assessors, which is expected in exercise trials. The risk of bias assessment can be visualised in Figs. 3.

The level of certainty of the evidence are presented in Table 2, Table 3, Table 4. In the comparison of HIIT vs MICT, evidence of a greater reduction in BMI, weight, and WC in the MICT group compared to the HIIT group was of low certainty. Certainty of evidence was also low for the greater reduction in HbA1c, triglycerides, FAI, and SHBG in the HIIT group compared to MICT. There was low certainty in the evidence showing no difference in HDL-C levels between the HIIT and MICT groups. All other outcomes in this comparison and the other comparisons (HIIT vs RT and MICT vs MICT ​+ ​RT) were of very low certainty.

## 4 Discussion

The results of this systematic review and meta-analysis provide a novel comparison of various exercise modalities for the management of PCOS. Although the effect of exercise vs no exercise has been previously examined in a PCOS population [5,8,9,22], this study aimed to clarify from the available literature whether one specific exercise programme may lead to greater improvements in outcomes compared to other exercise types and was not exclusively conducted in order to determine the effects of HIIT versus MICT. Five studies comparing HIIT, MICT and/or RT (±diet) were identified and included in the systematic review and meta-analysis, comprising a total of 216 individuals. The meta-analyses comparing HIIT vs MICT did not find any statistically significant differences in anthropometric, metabolic, or hormonal/reproductive outcomes, including BMI, body weight, WC, HbA1c, FBG, fasting insulin, HOMA-IR, HDL-C, LDL-C, triglycerides, FAI, SHBG, and testosterone. The certainty of evidence for these outcomes was rated as low or very low, meaning the true effect may be, or is probably, markedly different from the estimated effect, respectively [28]. A single study [29] reported improved menstrual regularity in participants in the HIIT group in comparison to MICT; this was ranked as very low certainty of evidence. There were no significant differences in any of the other outcomes between comparison groups (HIIT vs MICT, HIIT vs RT, and diet ​+ ​MICT ​+ ​RT vs diet ​+ ​MICT), and all were ranked as very low certainty of evidence.

Menstrual cycle characteristics are increasingly recognised to be associated with long-term health outcomes [33]. Irregular cycles are associated with type 2 diabetes, coronary heart disease, ovarian cancer, and premature mortality [33]; therefore, improving menstrual regularity is an important outcome to consider when examining exercise interventions in PCOS. Previous literature has demonstrated improved menstrual regularity with MICT [10,34]; however, in our study, Patten et al., 2022 [29] found that HIIT improved menstrual regularity significantly more than MICT. Benham et al., 2021 [32] found no significant difference in the percentage of participants in the HIIT and the MICT groups with regular menstrual cycles pre-intervention and in the last three months of intervention. This study also found that participants with improved menstrual regularity had hyperandrogenism and were overweight or obese [32]. Although menstrual irregularity is associated with a higher BMI [33], both studies found that the effects of exercise on menstrual cyclicity appeared to be independent of weight loss [29,32]. This finding is in line with the overall findings of Patten et al., 2022 [29] and Almenning et al., 2015 [31], who observed the benefits of exercise were independent of weight loss. None of the studies in our review [7,[29], [30], [31], [32]] found a significant difference in weight loss between exercise intervention groups.

One of the most common barriers to exercise reported in individuals with PCOS is a lack of time [35]. HIIT elicits similar improvements to MICT despite lower energy expenditure and less time commitment, an effect driven, in part, by concomitant improvements cardiorespiratory fitness through increased mitochondrial oxidative capacity and content [16]. Therefore, HIIT may potentially lead to higher compliance in those with busy schedules as it ameliorates the barrier of lack of time [16]. Reported adherence to the exercise programmes in the studies included in our review was high, despite only including participants with sedentary lifestyles. However, the intervention duration of these studies is short, and therefore no conclusion can be drawn regarding long-term compliance for such interventions. Other studies examining exercise intervention in a PCOS population have also reported high compliance rates [22], even though adherence to exercise intervention in clinical trials is generally considered low (34–36). When prescribing an exercise regime to PCOS patients, there are important practical considerations to keep in mind, such as the interest and enjoyment of the training programme, as this may help improve adherence to the programme [36].

### 4.1 Implications of the research

Exercise has been shown to improve cardiometabolic risk factors in patients with PCOS [9], including cardiorespiratory fitness, body composition, insulin resistance, and health-related quality of life [8]. Previous research has found that both HIIT and MICT, when compared to a non-exercise control, improved cardiorespiratory fitness; however, only MICT significantly improved waist circumference, which predicts cardiovascular risk more accurately than BMI [9]. For psychological outcomes, various exercise modalities improved anxiety, depression, and sexual function in individuals with PCOS [8,21], although HIIT was found to be the most effective; while RT did not significantly improve quality of life in comparison to HIIT or MICT [21]. This review found that there was no statistically significant difference between the exercise modalities in anthropometric, metabolic, and hormonal/reproductive outcomes for patients with PCOS. This finding is in line with previous research, as various exercise modalities were found to improve outcomes, but no single exercise modality outperformed others.

There remains a need for further research with larger sample sizes comparing multiple exercise modalities, matched for training volume, to allow for careful appraisal and to ascertain the true effect of each training programme. Future studies should consider the complexity of PCOS and the various phenotypes of the disease [37], aiming to understand how outcomes following exercise intervention may vary in these subgroups. Examining the individual-level factors underlying the response to exercise would allow a deeper understanding of the mechanisms by which exercise affects physiological pathways. Additionally, knowledge of these individual-level factors and their influence on outcomes would allow more precise exercise prescription on a patient-by-patient basis instead of a “one-size-fits-most” approach [38]. Studies with long-term follow-up are also required to determine adherence to various exercise modalities over a prolonged period. Broader outcome measures, including a focus on psychological outcomes such as body image, disordered eating behaviour, and sexual well-being, would provide a holistic assessment of the effects of exercise on patients with PCOS.

### 4.2 Strengths and limitations

The systematic review has limitations that should be considered when interpreting the results. 1; only five studies were included in this review, many of which had small sample sizes (total of 216 individuals), which may increase the likelihood of type I and II error. 2; this lack of available data limited our ability to directly compare the effectiveness of aerobic exercise to RT for relevant outcomes. Additionally, we were unable to determine the relative importance of exercise prescription variables such as intervention intensity, duration, and volume due to the variation in exercise protocols between studies. 3; supervised exercise sessions have been shown to be more effective than unsupervised exercise [39], but in the studies included in this review, most exercise sessions were unsupervised. However, most patients with PCOS may not be able to have supervised exercise sessions in their regular life and therefore, these results may be more in line with what would be expected outside of a clinical trial environment. 4; PCOS is a heterogenous condition with multiple phenotypes. Given the lack of available evidence, we were unable to conduct a sub-group analysis to determine how the outcomes of each exercise modality may vary with different PCOS phenotypes.

Notwithstanding these limitations, the studies included are RCTs, the study design most suited to determining causality. The systematic review was conducted in accordance with international guidelines and with a comprehensive search and rigorous methodological assessments. Our findings summarise the current evidence for exercise modalities for managing PCOS, highlighting the important gap in evidence and the need for future research in this area. These results will also directly inform the current update of the international evidence-based guidelines for the assessment and management of PCOS.

## 5 Conclusions

This systematic review and meta-analysis found low-level evidence that there were no statistically significant differences in anthropometric, metabolic and hormonal/reproductive outcomes between HIIT vs MICT. There were also no differences in any outcome following descriptive analyses of studies involving HIIT vs RT, and diet ​+ ​MICT ​+ ​RT vs diet ​+ ​MICT. Our findings suggest that there are no differences in these exercise modalities for managing PCOS, however, as noted above, the certainty of evidence was very low largely due to small number of studies included which limited our ability to conduct more detailed analyses based on exercise modalities. Based on these results, patients may select their preferred method of training, leading to a more individualised exercise prescription, rather than a “one-size-fits-most” approach. As long-term adherence to exercise programmes is a clinically relevant issue, flexibility in the choice of exercise type could potentially lead to improved compliance. However, given the small number of studies and sample size of five studies and 216 participants, the limited exercise modalities identified, as well as the low to very low certainty of evidence, further research is required to establish which exercise modalities are most effective in the management of specific health outcomes in PCOS.

## Author contributions

GEC was involved in the study selection and drafting of the manuscript. XDB was involved in the study selection, data extraction and editing of the manuscript. RKP was involved in the study selection and editing of the manuscript. AM was involved in the study design and editing of the manuscript. CTT was involved in the editing of the manuscript. LP was involved in the study design and editing of the manuscript. LMR was involved in the study design and editing of the manuscript. AHL was involved in the study design and editing of the manuscript. HT was involved in the study design and editing of the manuscript. AS was involved in the search strategy, study selection, data extraction, statistical analysis, data interpretation, risk of bias and study quality assessment, and drafting of the manuscript. All authors have read and agreed to the published version of the manuscript.

## Funding and support

HT and AM are supported by Australian 10.13039/501100000925National Health and Medical Research Council (NHMRC) fellowships, and the guideline and associated processes are supported by an NHMRC Centre for Research Excellence (1171592). LMR is supported in part by Pennington/Louisiana Nutrition Obesity Research Center (P30 DK072476) and various National Institute of Health investigator grants (R01 NR017644, R01 DK124806, 5U24 AG047121, U01AG073204, UG1HD107696). AS is supported by Western Sydney University through a Research Support Program Fellowship.

## Declaration of competing interest

The authors report no conflicts of interest.

## Figures and Tables

**Table S1:** Database search Strategy

OVID Medline, All EBM, PsychInfo, EMBASE (results ​= ​4618)	
1	exp polycystic ovary syndrome/
2	polycystic ovar∗.mp.
3	PCO∗.mp.
4	(stein-leventhal or leventhal).mp.
5	anovulation/
6	anovulat∗.mp.
7	oligo-ovulat∗.mp.
8	oligoovulat∗.mp.
9	(ovar∗ adj5 (sclerocystic or polycystic or poly-cystic or degenerat∗ or hyperandrogen∗ or hyper-androgen∗)).mp.
10	poly-cystic ovar∗.mp.
11	or/1-10
12	exercise∗.mp.
13	exercise therapy.mp.
14	exertion.mp.
15	physical fitness.mp.
16	physical activit∗.mp.
17	physical performance.mp.
18	sport∗.mp.
19	(strength adj2 training).mp.
20	resistance training.mp.
21	(aerobic∗ adj2 training).mp.
22	(endurance adj training).mp.
23	physical training.mp.
24	(strength∗ adj2 exercise∗).mp.
25	(weight-bearing adj2 exercise∗).mp.
26	(Resistance adj2 exercise∗).mp.
27	(Aerobic∗ adj2 exercise∗).mp.
28	(Endurance adj2 exercise∗).mp.
29	(Physical adj2 exercise∗).mp.
30	fit∗.mp.
31	bicycle∗.mp.
32	cycl∗.mp.
33	run∗.mp.
34	swim∗.mp.
35	walk∗.mp.
36	jog∗.mp.
37	train∗.mp.
38	gym∗.mp.
39	aqua-aerobics.mp.
40	pilates.mp.
41	yoga.mp.
42	danc∗.mp.
43	exp exercise/
44	exp exercise therapy/
45	physical exertion/
46	exp sports/
47	exp physical endurance/
48	or/12-47
49	search$.tw. or meta-analysis.mp. or meta-analysis.pt. or review.pt. or di.tw,kw. or associated.tw.
50	clinical trial.mp. or clinical trial.pt. or random.mp. or tu.xs.
51	49 or 50
52	11 and 48 and 51
53	limit 52 to (english language and humans and yr ​= ​"2017 -Current")

**Table S2:** Excluded studies following full-text review with rationale for exclusion

Study reference	Rationale
Al-Eisa et al., 2017	Wrong comparator
Benham et al., 2020	Conference abstract
Elbandrawy et al., 2022	Wrong comparator
Furtado et al., 2020	Conference abstract
Hansen et al., 2020	Wrong patient population
Jerobin et al., 2021	Wrong comparator
Kazemi et al., 2018	Wrong comparator
Kiel et al., 2022	Wrong comparator
Kiel et al., 2022	Wrong comparator
Lara et al., 2018	Conference abstract
Li et al., 2019	Wrong intervention
Li et al., 2020	Wrong intervention
Lionett et al., 2020	Wrong comparator
Patel et al., 2018	Wrong intervention
Ramanjaneya et al., 2018	Wrong study design
Scott et al., 2017	Wrong study design
Shalini et al., 2020	Wrong intervention
Veena Kirthika et al., 2019	Wrong comparator
Woodward et al., 2022	Wrong comparator
